# Bis([μ-bis­(diphenyl­phosphino)methane-1:2κ^2^ *P*:*P*′]nona­carbonyl-1κ^3^ *C*,2κ^3^ *C*,3κ^3^ *C*-{tris­[4-(methyl­sulfan­yl)phen­yl]arsine-3κ*As*}-*triangulo*-triruthenium(0)) dichloro­methane monosolvate

**DOI:** 10.1107/S160053681003093X

**Published:** 2010-08-21

**Authors:** Omar bin Shawkataly, Imthyaz Ahmed Khan, Siti Syaida Sirat, Chin Sing Yeap, Hoong-Kun Fun

**Affiliations:** a Chemical Sciences Programme, School of Distance Education, Universiti Sains Malaysia, 11800 USM, Penang, Malaysia; b X-ray Crystallography Unit, School of Physics, Universiti Sains Malaysia, 11800 USM, Penang, Malaysia

## Abstract

The asymmetric unit of the title *triangulo*-triruthenium compound, 2[Ru_3_(C_21_H_21_AsS_3_)(C_25_H_22_P_2_)(CO)_9_]·CH_2_Cl_2_, consists of one *triangulo*-triruthenium complex mol­ecule and one half of a dichloro­methane mol­ecule which lies across a crystallographic inversion center, leading to the disorder of this mol­ecule over two positions of equal occupancy. The bis­(diphenyl­phosphino)methane ligand bridges an Ru—Ru bond and the monodentate arsine ligand bonds to the third Ru atom. Both the arsine and phosphine ligands are equatorial with respect to the Ru_3_ triangle. Each Ru atom carries one equatorial and two axial terminal carbonyl ligands. The three arsine-substituted benzene rings make dihedral angles of 82.69 (9), 70.43 (9) and 89.45 (9)° with each other. The dihedral angles between the two benzene rings are 85.14 (11) and 77.61 (10)° for the two diphenyl­phosphino groups. In the crystal packing, mol­ecules are linked together into dimers *via* inter­molecular C—H⋯O hydrogen bonds and these dimers are stacked along the *a* axis. Weak inter­molecular C—H⋯π inter­actions are also present.

## Related literature

For general background to *triangulo*-triruthenium derivatives, see: Bruce *et al.* (1985 ▶); Bruce, Liddell, Hughes *et al.* (1988 ▶); Bruce, Liddell, Shawkataly *et al.* (1988 ▶). For related structures, see: Shawkataly *et al.* (1998 ▶, 2004 ▶, 2010 ▶, 2010*a* ▶,*b* ▶). For the synthesis of bis­(diphenyl­phosphino)methane, see: Bruce *et al.* (1983 ▶). For the stability of the temperature controller used in the data collection, see: Cosier & Glazer (1986 ▶).
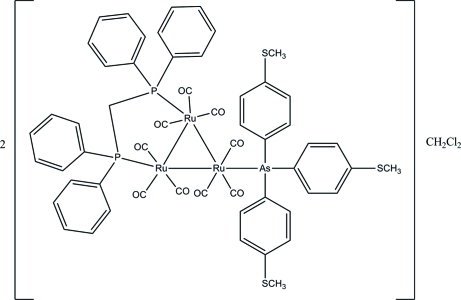


## Experimental

### 

#### Crystal data

2[Ru_3_(C_21_H_21_AsS_3_)(C_25_H_22_P_2_)(CO)_9_]·CH_2_Cl_2_
*M* *_r_* = 2853.21
Triclinic, 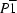

*a* = 10.8077 (1) Å
*b* = 12.6180 (2) Å
*c* = 20.9478 (3) Å
α = 94.539 (1)°
β = 98.228 (1)°
γ = 103.212 (1)°
*V* = 2733.83 (6) Å^3^
*Z* = 1
Mo *K*α radiation
μ = 1.70 mm^−1^
*T* = 100 K
0.36 × 0.31 × 0.15 mm

#### Data collection

Bruker SMART APEXII CCD area-detector diffractometer
Absorption correction: multi-scan (*SADABS*; Bruker, 2009 ▶) *T*_min_ = 0.583, *T*_max_ = 0.786
305620 measured reflections
24183 independent reflections
20753 reflections with *I* > 2σ(*I*)
*R*_int_ = 0.036

#### Refinement

*R*[*F* ^2^ > 2σ(*F* ^2^)] = 0.031
*wR*(*F* ^2^) = 0.070
*S* = 1.08
24183 reflections
679 parameters
H-atom parameters constrained
Δρ_max_ = 1.50 e Å^−3^
Δρ_min_ = −1.34 e Å^−3^

### 

Data collection: *APEX2* (Bruker, 2009 ▶); cell refinement: *SAINT* (Bruker, 2009 ▶); data reduction: *SAINT*; program(s) used to solve structure: *SHELXTL* (Sheldrick, 2008 ▶); program(s) used to refine structure: *SHELXTL*; molecular graphics: *SHELXTL*; software used to prepare material for publication: *SHELXTL* and *PLATON* (Spek, 2009 ▶).

## Supplementary Material

Crystal structure: contains datablocks global, I. DOI: 10.1107/S160053681003093X/is2583sup1.cif

Structure factors: contains datablocks I. DOI: 10.1107/S160053681003093X/is2583Isup2.hkl

Additional supplementary materials:  crystallographic information; 3D view; checkCIF report

## Figures and Tables

**Table 1:** Hydrogen-bond geometry (Å, °) *Cg*1, *Cg*2, *Cg*3 and *Cg*4 are the centroids of the C14–C19, C26–C31, C1–C6 and C32–C37 benzene rings, respectively.

*D*—H⋯*A*	*D*—H	H⋯*A*	*D*⋯*A*	*D*—H⋯*A*
C46—H46*C*⋯O4^i^	0.96	2.59	3.401 (3)	142
C11—H11*A*⋯*Cg*1^ii^	0.93	2.91	3.640 (2)	137
C18—H18*A*⋯*Cg*2^iii^	0.93	2.96	3.652 (2)	133
C36—H36*A*⋯*Cg*3^iv^	0.93	2.81	3.687 (2)	157
C45—H45*B*⋯*Cg*4^v^	0.96	2.90	3.760 (3)	150

Symmetry codes: (i) 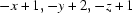; (ii) 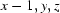; (iii) 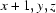; (iv) 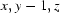; (v) 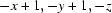.

**Fig. 1.**
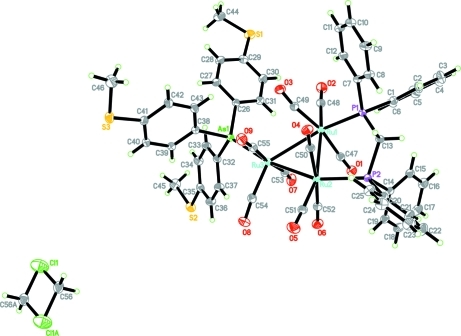
The molecular structure of the title compound with 50% probability ellipsoids for non-H atoms. Atoms with suffix A are generated by the symmetry operation (1 - x, -y, 1 - z).

**Fig. 2.**
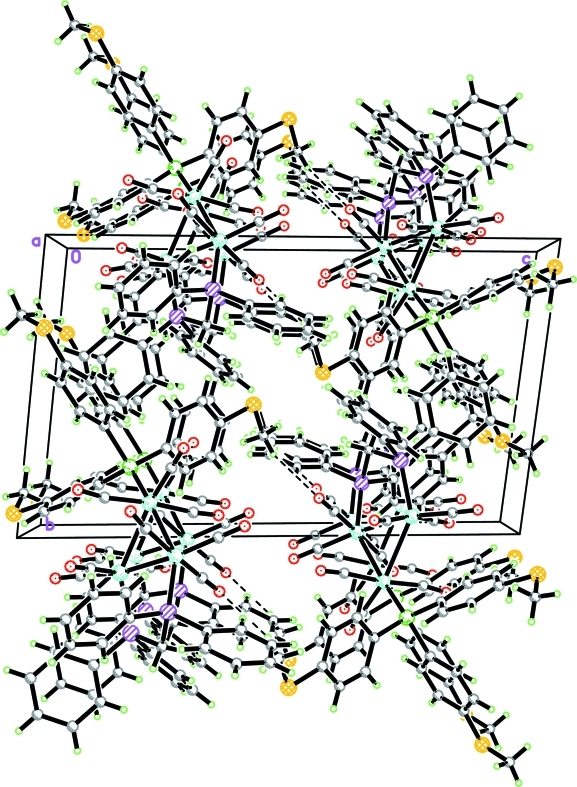
The crystal packing of the title compound, viewed down the a axis, showing the molecules link into dimers stacking along a axis. Intermolecular hydrogen bonds are shown as dashed lines and solvent molecule have been omitted for clarity.

## supplementary crystallographic information

### Comment

*Triangulo*-triruthenium clusters are known for their interesting structural variations and related catalytic activity. A large number of substituted derivatives, Ru_3_(CO)_12-n_*L*_n_ (*L* = group 15 ligand) have been reported (Bruce, Liddell, Hughes *et al.*, 1988; Bruce, Liddell, Shawkataly *et al.*, 1988; Bruce *et al.*, 1985). As part of our study on the substitution of transition metal-carbonyl clusters with mixed-ligand complexes, we have published several structures of *triangulo*-triruthenium-carbonyl clusters containing mixed P/As and P/Sb ligands (Shawkataly *et al.*, 1998, 2004; Shawkataly, Khan, Yeap & Fun, 2010*a*, *b*; Shawkataly, Khan, Sirat *et al.*, 2010). Herein we report the synthesis and structure of the title compound.

The asymmetry unit consists of one molecule of *triangulo*-triruthenium complex and half a molecule of dichloromethane solvent (Fig. 1). The dichloromethane solvent lies across a crystallographic inversion center leading to disorder of this solvent molecule over two positions. The geometric parameters of title compound are comparable to those found in related structures (Shawkataly, Khan, Yeap & Fun, 2010*a*,*b*; Shawkataly, Khan, Sirat *et al.*, 2010). The bis(diphenylphosphino)methane ligand bridges the Ru1—Ru2 bond and the monodentate arsine ligand bonds to the Ru3 atom. Both the arsine and phosphine ligands are equatorial with respect to the Ru_3_ triangle. Additionally, each Ru atom carries one equatorial and two axial terminal carbonyl ligands. The three arsine-substituted benzene rings make dihedral angles (C26–C31/C32–C37, C26–C31/C38–C43 and C32–C37/C38–C43) of 82.69 (9), 70.43 (9) and 89.45 (9)° with each other respectively. The dihedral angles between the two benzene rings (C1–C6/C7–C12 and C14–C19/C20–C25) are 85.14 (11) and 77.61 (10)° for the two diphenylphosphino groups respectively. The methylsulfanyl groups are nearly coplanar with the attached benzene rings [torsion angles C44–S1–C29–C28 = -8.6 (2), C45–S2–C35–C34 = -8.6 (2) and C46–S3–C41–C42 = 14.6 (2)°].

In the crystal packing, the molecules are linked together into dimers *via* intermolecular C46—H46C···O4 hydrogen bonds (Table 1) and these dimers are stacked along the *a* axis (Fig. 2). Weak intermolecular C—H···π interactions (Table 1) further stabilize the crystal structure.

### Experimental

All manipulations were performed under a dry oxygen-free nitrogen atmosphere using standard Schlenk techniques. All solvents were dried over sodium and distilled from sodium benzophenone ketyl under dry oxygen-free nitrogen. Tris(4-(methylsulfanyl)phenyl)arsine was prepared from arsenic trichloride and 4-(methylsulfanyl)phenylmagnesium bromide in tetrahydrofuran and bis(diphenylphosphino)methane (Bruce *et al.*, 1983) was prepared by reported procedure. The title compound was obtained by refluxing equimolar quantities of Ru_3_(CO)_10_(µ-Ph_2_PCH_2_PPh_2_) and tris(4-(methylsulfanyl)phenyl)arsine in hexane under nitrogen atmosphere. Crystals suitable for X-ray diffraction were grown by slow solvent / solvent diffusion of CH_3_OH into CH_2_Cl_2_.

### Refinement

All hydrogen atoms were positioned geometrically and refined using a riding model with C—H = 0.93–0.97 Å and *U*_iso_(H) = 1.2 or 1.5 *U*_eq_(C). Rotating group model was applied for the methyl groups. The highest peak and deepest hole in the difference Fourier map are located 0.88 and 0.64 Å, respectively, from atoms C45 and C11.

### Crystal data

**Table:**

2[Ru_3_(C_21_H_21_AsS_3_)(C_25_H_22_P_2_)(CO)_9_]·CH_2_Cl_2_	*Z* = 1
*M**_r_* = 2853.21	*F*(000) = 1418
Triclinic, *P*1	*D*_x_ = 1.733 Mg m^−^^3^
Hall symbol: -P 1	Mo *K*α radiation, λ = 0.71073 Å
*a* = 10.8077 (1) Å	Cell parameters from 9102 reflections
*b* = 12.6180 (2) Å	θ = 2.3–34.9°
*c* = 20.9478 (3) Å	µ = 1.70 mm^−^^1^
α = 94.539 (1)°	*T* = 100 K
β = 98.228 (1)°	Block, purple
γ = 103.212 (1)°	0.36 × 0.31 × 0.15 mm
*V* = 2733.83 (6) Å^3^	

### Data collection

**Table:**

Bruker SMART APEXII CCD area-detector diffractometer	24183 independent reflections
Radiation source: fine-focus sealed tube	20753 reflections with *I* > 2σ(*I*)
graphite	*R*_int_ = 0.036
φ and ω scans	θ_max_ = 35.2°, θ_min_ = 1.7°
Absorption correction: multi-scan (*SADABS*; Bruker, 2009)	*h* = −17→17
*T*_min_ = 0.583, *T*_max_ = 0.786	*k* = −20→20
305620 measured reflections	*l* = −32→33

### Refinement

**Table:**

Refinement on *F*^2^	Primary atom site location: structure-invariant direct methods
Least-squares matrix: full	Secondary atom site location: difference Fourier map
*R*[*F*^2^ > 2σ(*F*^2^)] = 0.031	Hydrogen site location: inferred from neighbouring sites
*wR*(*F*^2^) = 0.070	H-atom parameters constrained
*S* = 1.08	*w* = 1/[σ^2^(*F*_o_^2^) + (0.021*P*)^2^ + 4.6125*P*] where *P* = (*F*_o_^2^ + 2*F*_c_^2^)/3
24183 reflections	(Δ/σ)_max_ = 0.005
679 parameters	Δρ_max_ = 1.50 e Å^−^^3^
0 restraints	Δρ_min_ = −1.34 e Å^−^^3^

### Special details

**Table:**

Experimental. The crystal was placed in the cold stream of an Oxford Cryosystems Cobra open-flow nitrogen cryostat (Cosier & Glazer, 1986) operating at 100.0 (1) K.
Geometry. All e.s.d.'s (except the e.s.d. in the dihedral angle between two l.s. planes) are estimated using the full covariance matrix. The cell e.s.d.'s are taken into account individually in the estimation of e.s.d.'s in distances, angles and torsion angles; correlations between e.s.d.'s in cell parameters are only used when they are defined by crystal symmetry. An approximate (isotropic) treatment of cell e.s.d.'s is used for estimating e.s.d.'s involving l.s. planes.
Refinement. Refinement of *F*^2^ against ALL reflections. The weighted *R*-factor *wR* and goodness of fit *S* are based on *F*^2^, conventional *R*-factors *R* are based on *F*, with *F* set to zero for negative *F*^2^. The threshold expression of *F*^2^ > σ(*F*^2^) is used only for calculating *R*-factors(gt) *etc*. and is not relevant to the choice of reflections for refinement. *R*-factors based on *F*^2^ are statistically about twice as large as those based on *F*, and *R*- factors based on ALL data will be even larger.

### Fractional atomic coordinates and isotropic or equivalent isotropic displacement parameters (Å^2^)

**Table:**

	*x*	*y*	*z*	*U*_iso_*/*U*_eq_	Occ. (<1)
Ru1	0.789717 (12)	1.074563 (10)	0.230124 (6)	0.01282 (2)	
Ru2	0.955747 (12)	1.014066 (10)	0.331223 (6)	0.01201 (2)	
Ru3	0.732672 (12)	0.856245 (10)	0.269877 (6)	0.01296 (2)	
As1	0.515421 (16)	0.749783 (13)	0.220967 (8)	0.01364 (3)	
P1	0.87864 (4)	1.26009 (3)	0.26392 (2)	0.01301 (7)	
P2	1.10728 (4)	1.17749 (3)	0.33137 (2)	0.01262 (7)	
S1	0.14677 (5)	0.91123 (5)	−0.00329 (2)	0.02511 (9)	
S2	0.53088 (5)	0.31201 (4)	0.03880 (2)	0.02334 (9)	
S3	0.17287 (5)	0.55384 (4)	0.43536 (2)	0.02273 (9)	
O1	0.99444 (14)	1.04100 (12)	0.14713 (7)	0.0213 (2)	
O2	0.61722 (15)	1.10054 (13)	0.10736 (8)	0.0279 (3)	
O3	0.56621 (14)	1.07036 (13)	0.30652 (8)	0.0258 (3)	
O4	0.80979 (13)	1.13552 (12)	0.41588 (7)	0.0218 (3)	
O5	1.06190 (17)	0.91808 (14)	0.44992 (8)	0.0303 (3)	
O6	1.07660 (14)	0.86993 (12)	0.24560 (7)	0.0240 (3)	
O7	0.82395 (15)	0.81171 (12)	0.14058 (7)	0.0254 (3)	
O8	0.85901 (15)	0.68341 (12)	0.32256 (8)	0.0260 (3)	
O9	0.63093 (15)	0.90166 (12)	0.39677 (7)	0.0247 (3)	
C1	0.92078 (17)	1.35527 (14)	0.20399 (9)	0.0167 (3)	
C2	0.96341 (19)	1.46840 (15)	0.22417 (10)	0.0225 (3)	
H2A	0.9733	1.4940	0.2679	0.027*	
C3	0.9908 (2)	1.54212 (17)	0.17939 (12)	0.0316 (5)	
H3A	1.0196	1.6168	0.1932	0.038*	
C4	0.9752 (3)	1.50426 (19)	0.11383 (13)	0.0355 (5)	
H4A	0.9941	1.5536	0.0838	0.043*	
C5	0.9316 (2)	1.39300 (19)	0.09312 (11)	0.0308 (4)	
H5A	0.9198	1.3681	0.0492	0.037*	
C6	0.9052 (2)	1.31822 (16)	0.13816 (9)	0.0215 (3)	
H6A	0.8773	1.2436	0.1242	0.026*	
C7	0.77484 (16)	1.33157 (13)	0.30306 (8)	0.0155 (3)	
C8	0.81545 (18)	1.40218 (15)	0.36071 (9)	0.0192 (3)	
H8A	0.8990	1.4122	0.3829	0.023*	
C9	0.73212 (19)	1.45786 (16)	0.38536 (9)	0.0215 (3)	
H9A	0.7598	1.5044	0.4240	0.026*	
C10	0.60814 (19)	1.44401 (15)	0.35230 (10)	0.0216 (3)	
H10A	0.5525	1.4812	0.3688	0.026*	
C11	0.56677 (18)	1.37492 (16)	0.29474 (10)	0.0223 (3)	
H11A	0.4836	1.3663	0.2725	0.027*	
C12	0.64908 (17)	1.31841 (15)	0.27014 (9)	0.0190 (3)	
H12A	0.6206	1.2716	0.2316	0.023*	
C13	1.02687 (16)	1.29086 (13)	0.32548 (8)	0.0150 (3)	
H13A	1.0870	1.3544	0.3151	0.018*	
H13B	1.0051	1.3099	0.3675	0.018*	
C14	1.20348 (16)	1.18633 (14)	0.26653 (8)	0.0149 (3)	
C15	1.20290 (17)	1.26488 (15)	0.22314 (9)	0.0186 (3)	
H15A	1.1591	1.3193	0.2295	0.022*	
C16	1.26785 (19)	1.26183 (18)	0.17042 (10)	0.0243 (4)	
H16A	1.2673	1.3144	0.1418	0.029*	
C17	1.33347 (19)	1.18084 (18)	0.16030 (10)	0.0246 (4)	
H17A	1.3747	1.1780	0.1244	0.030*	
C18	1.33699 (18)	1.10422 (17)	0.20416 (9)	0.0216 (3)	
H18A	1.3822	1.0508	0.1981	0.026*	
C19	1.27308 (17)	1.10715 (14)	0.25718 (9)	0.0174 (3)	
H19A	1.2767	1.0561	0.2866	0.021*	
C20	1.22350 (16)	1.22969 (13)	0.40601 (8)	0.0150 (3)	
C21	1.35464 (18)	1.27053 (16)	0.40655 (9)	0.0208 (3)	
H21A	1.3871	1.2704	0.3678	0.025*	
C22	1.4378 (2)	1.31169 (18)	0.46514 (10)	0.0261 (4)	
H22A	1.5255	1.3384	0.4652	0.031*	
C23	1.3905 (2)	1.31285 (17)	0.52300 (10)	0.0249 (4)	
H23A	1.4460	1.3405	0.5619	0.030*	
C24	1.2597 (2)	1.27251 (17)	0.52274 (9)	0.0229 (3)	
H24A	1.2276	1.2738	0.5616	0.027*	
C25	1.17665 (18)	1.23034 (15)	0.46504 (9)	0.0194 (3)	
H25A	1.0894	1.2023	0.4655	0.023*	
C26	0.39948 (16)	0.80880 (13)	0.16213 (8)	0.0155 (3)	
C27	0.26723 (17)	0.78662 (15)	0.16155 (9)	0.0193 (3)	
H27A	0.2323	0.7505	0.1943	0.023*	
C28	0.18649 (18)	0.81801 (16)	0.11239 (9)	0.0207 (3)	
H28A	0.0983	0.8037	0.1127	0.025*	
C29	0.23805 (18)	0.87091 (15)	0.06264 (9)	0.0188 (3)	
C30	0.37130 (19)	0.89558 (16)	0.06400 (9)	0.0207 (3)	
H30A	0.4066	0.9324	0.0316	0.025*	
C31	0.45127 (17)	0.86529 (14)	0.11362 (9)	0.0186 (3)	
H31A	0.5399	0.8828	0.1145	0.022*	
C32	0.51489 (17)	0.61810 (13)	0.16580 (8)	0.0161 (3)	
C33	0.41482 (18)	0.57361 (14)	0.11431 (9)	0.0199 (3)	
H33A	0.3458	0.6061	0.1068	0.024*	
C34	0.41688 (19)	0.48096 (15)	0.07386 (9)	0.0213 (3)	
H34A	0.3500	0.4527	0.0393	0.026*	
C35	0.51893 (19)	0.43051 (14)	0.08509 (9)	0.0187 (3)	
C36	0.61985 (19)	0.47521 (15)	0.13645 (9)	0.0212 (3)	
H36A	0.6888	0.4427	0.1441	0.025*	
C37	0.61745 (18)	0.56789 (15)	0.17616 (9)	0.0205 (3)	
H37A	0.6852	0.5970	0.2102	0.025*	
C38	0.40518 (16)	0.69247 (13)	0.28205 (8)	0.0154 (3)	
C39	0.38059 (17)	0.58160 (14)	0.29089 (9)	0.0173 (3)	
H39A	0.4118	0.5337	0.2651	0.021*	
C40	0.30962 (17)	0.54248 (14)	0.33805 (9)	0.0181 (3)	
H40A	0.2961	0.4689	0.3445	0.022*	
C41	0.25826 (17)	0.61239 (14)	0.37597 (9)	0.0169 (3)	
C42	0.28079 (18)	0.72304 (15)	0.36634 (9)	0.0199 (3)	
H42A	0.2460	0.7702	0.3907	0.024*	
C43	0.35491 (18)	0.76292 (14)	0.32052 (9)	0.0194 (3)	
H43A	0.3714	0.8371	0.3153	0.023*	
C44	−0.0172 (2)	0.8498 (2)	0.00396 (11)	0.0289 (4)	
H44A	−0.0741	0.8627	−0.0326	0.043*	
H44B	−0.0369	0.8816	0.0433	0.043*	
H44C	−0.0281	0.7724	0.0051	0.043*	
C45	0.3761 (2)	0.26979 (19)	−0.01170 (11)	0.0306 (4)	
H45A	0.3674	0.1993	−0.0352	0.046*	
H45B	0.3677	0.3222	−0.0419	0.046*	
H45C	0.3100	0.2653	0.0148	0.046*	
C46	0.0931 (3)	0.65660 (19)	0.46065 (12)	0.0321 (5)	
H46A	0.0323	0.6268	0.4877	0.048*	
H46B	0.0486	0.6793	0.4231	0.048*	
H46C	0.1558	0.7186	0.4846	0.048*	
C47	0.92190 (17)	1.05241 (14)	0.18044 (8)	0.0171 (3)	
C48	0.67963 (17)	1.08893 (14)	0.15414 (9)	0.0185 (3)	
C49	0.65165 (17)	1.06542 (15)	0.28060 (9)	0.0186 (3)	
C50	0.85955 (16)	1.08903 (14)	0.38234 (8)	0.0162 (3)	
C51	1.02419 (17)	0.95376 (14)	0.40485 (9)	0.0179 (3)	
C52	1.02916 (17)	0.92561 (14)	0.27472 (9)	0.0176 (3)	
C53	0.79256 (17)	0.83725 (15)	0.18851 (9)	0.0188 (3)	
C54	0.80506 (18)	0.74630 (14)	0.30340 (9)	0.0184 (3)	
C55	0.67171 (17)	0.89014 (14)	0.35021 (9)	0.0180 (3)	
Cl1	0.38996 (8)	0.00599 (6)	0.45153 (4)	0.04883 (17)	
C56	0.5335 (4)	0.0655 (3)	0.4924 (2)	0.0284 (8)	0.50
H56A	0.5271	0.1322	0.5156	0.034*	0.50
H56B	0.5897	0.0856	0.4614	0.034*	0.50

### Atomic displacement parameters (Å^2^)

**Table:**

	*U*^11^	*U*^22^	*U*^33^	*U*^12^	*U*^13^	*U*^23^
Ru1	0.01295 (5)	0.01199 (5)	0.01326 (5)	0.00315 (4)	0.00049 (4)	0.00250 (4)
Ru2	0.01166 (5)	0.01287 (5)	0.01219 (5)	0.00396 (4)	0.00212 (4)	0.00244 (4)
Ru3	0.01298 (5)	0.01205 (5)	0.01406 (5)	0.00286 (4)	0.00259 (4)	0.00265 (4)
As1	0.01331 (7)	0.01265 (7)	0.01545 (7)	0.00372 (5)	0.00291 (6)	0.00193 (5)
P1	0.01247 (16)	0.01252 (16)	0.01434 (17)	0.00372 (13)	0.00158 (13)	0.00251 (13)
P2	0.01157 (16)	0.01369 (16)	0.01305 (17)	0.00384 (13)	0.00196 (13)	0.00208 (13)
S1	0.0250 (2)	0.0332 (2)	0.0187 (2)	0.01099 (19)	0.00041 (17)	0.00628 (17)
S2	0.0308 (2)	0.01854 (18)	0.0215 (2)	0.00746 (17)	0.00672 (18)	−0.00113 (15)
S3	0.0260 (2)	0.02171 (19)	0.0213 (2)	0.00338 (16)	0.00942 (17)	0.00470 (16)
O1	0.0219 (6)	0.0247 (6)	0.0178 (6)	0.0056 (5)	0.0043 (5)	0.0032 (5)
O2	0.0261 (7)	0.0312 (7)	0.0244 (7)	0.0054 (6)	−0.0043 (6)	0.0105 (6)
O3	0.0198 (6)	0.0313 (7)	0.0323 (8)	0.0116 (5)	0.0093 (6)	0.0142 (6)
O4	0.0199 (6)	0.0250 (6)	0.0213 (6)	0.0074 (5)	0.0050 (5)	−0.0015 (5)
O5	0.0349 (8)	0.0353 (8)	0.0243 (7)	0.0141 (7)	0.0021 (6)	0.0136 (6)
O6	0.0220 (6)	0.0252 (6)	0.0263 (7)	0.0090 (5)	0.0066 (5)	−0.0024 (5)
O7	0.0267 (7)	0.0261 (7)	0.0210 (6)	0.0005 (5)	0.0081 (5)	−0.0013 (5)
O8	0.0302 (7)	0.0237 (6)	0.0267 (7)	0.0123 (6)	0.0021 (6)	0.0068 (5)
O9	0.0306 (7)	0.0244 (6)	0.0213 (6)	0.0073 (5)	0.0105 (6)	0.0032 (5)
C1	0.0154 (7)	0.0162 (6)	0.0194 (7)	0.0042 (5)	0.0033 (6)	0.0054 (5)
C2	0.0240 (8)	0.0159 (7)	0.0256 (9)	0.0004 (6)	0.0034 (7)	0.0048 (6)
C3	0.0351 (11)	0.0184 (8)	0.0410 (12)	0.0015 (8)	0.0098 (9)	0.0103 (8)
C4	0.0466 (14)	0.0281 (10)	0.0373 (12)	0.0084 (9)	0.0181 (10)	0.0187 (9)
C5	0.0428 (13)	0.0304 (10)	0.0239 (9)	0.0114 (9)	0.0127 (9)	0.0110 (8)
C6	0.0264 (9)	0.0203 (7)	0.0200 (8)	0.0069 (6)	0.0069 (7)	0.0065 (6)
C7	0.0140 (6)	0.0147 (6)	0.0191 (7)	0.0053 (5)	0.0035 (5)	0.0035 (5)
C8	0.0178 (7)	0.0207 (7)	0.0197 (8)	0.0080 (6)	0.0009 (6)	0.0006 (6)
C9	0.0234 (8)	0.0225 (8)	0.0208 (8)	0.0108 (7)	0.0034 (6)	0.0005 (6)
C10	0.0204 (8)	0.0204 (7)	0.0274 (9)	0.0096 (6)	0.0071 (7)	0.0034 (6)
C11	0.0150 (7)	0.0219 (8)	0.0314 (10)	0.0075 (6)	0.0034 (7)	0.0033 (7)
C12	0.0158 (7)	0.0185 (7)	0.0227 (8)	0.0056 (6)	0.0011 (6)	0.0015 (6)
C13	0.0136 (6)	0.0137 (6)	0.0174 (7)	0.0041 (5)	0.0001 (5)	0.0011 (5)
C14	0.0119 (6)	0.0182 (7)	0.0148 (7)	0.0035 (5)	0.0027 (5)	0.0030 (5)
C15	0.0161 (7)	0.0218 (7)	0.0195 (8)	0.0052 (6)	0.0043 (6)	0.0068 (6)
C16	0.0215 (8)	0.0327 (10)	0.0208 (8)	0.0057 (7)	0.0069 (7)	0.0115 (7)
C17	0.0197 (8)	0.0371 (10)	0.0181 (8)	0.0057 (7)	0.0079 (6)	0.0045 (7)
C18	0.0176 (8)	0.0274 (8)	0.0212 (8)	0.0073 (6)	0.0057 (6)	0.0000 (7)
C19	0.0151 (7)	0.0201 (7)	0.0183 (7)	0.0052 (6)	0.0051 (6)	0.0031 (6)
C20	0.0157 (7)	0.0150 (6)	0.0138 (6)	0.0042 (5)	0.0003 (5)	0.0012 (5)
C21	0.0155 (7)	0.0264 (8)	0.0188 (8)	0.0018 (6)	0.0012 (6)	0.0047 (6)
C22	0.0183 (8)	0.0308 (9)	0.0241 (9)	−0.0009 (7)	−0.0022 (7)	0.0033 (7)
C23	0.0263 (9)	0.0256 (9)	0.0189 (8)	0.0051 (7)	−0.0054 (7)	−0.0015 (7)
C24	0.0267 (9)	0.0270 (9)	0.0151 (7)	0.0089 (7)	0.0012 (6)	0.0002 (6)
C25	0.0181 (7)	0.0234 (8)	0.0163 (7)	0.0052 (6)	0.0028 (6)	0.0009 (6)
C26	0.0154 (7)	0.0143 (6)	0.0173 (7)	0.0048 (5)	0.0022 (5)	0.0019 (5)
C27	0.0172 (7)	0.0226 (8)	0.0208 (8)	0.0075 (6)	0.0052 (6)	0.0071 (6)
C28	0.0163 (7)	0.0272 (8)	0.0205 (8)	0.0081 (6)	0.0038 (6)	0.0050 (6)
C29	0.0211 (8)	0.0197 (7)	0.0160 (7)	0.0074 (6)	0.0013 (6)	0.0000 (6)
C30	0.0213 (8)	0.0235 (8)	0.0177 (8)	0.0049 (6)	0.0041 (6)	0.0052 (6)
C31	0.0167 (7)	0.0192 (7)	0.0204 (8)	0.0044 (6)	0.0043 (6)	0.0028 (6)
C32	0.0174 (7)	0.0139 (6)	0.0172 (7)	0.0044 (5)	0.0034 (6)	0.0014 (5)
C33	0.0208 (8)	0.0166 (7)	0.0221 (8)	0.0060 (6)	0.0015 (6)	−0.0002 (6)
C34	0.0237 (8)	0.0186 (7)	0.0195 (8)	0.0045 (6)	0.0001 (6)	−0.0011 (6)
C35	0.0246 (8)	0.0160 (7)	0.0167 (7)	0.0048 (6)	0.0076 (6)	0.0025 (5)
C36	0.0213 (8)	0.0213 (8)	0.0223 (8)	0.0092 (6)	0.0030 (6)	−0.0007 (6)
C37	0.0202 (8)	0.0199 (7)	0.0217 (8)	0.0079 (6)	0.0010 (6)	−0.0007 (6)
C38	0.0146 (7)	0.0152 (6)	0.0168 (7)	0.0043 (5)	0.0030 (5)	0.0024 (5)
C39	0.0168 (7)	0.0146 (6)	0.0214 (8)	0.0043 (5)	0.0043 (6)	0.0036 (5)
C40	0.0169 (7)	0.0152 (6)	0.0223 (8)	0.0032 (5)	0.0036 (6)	0.0041 (6)
C41	0.0152 (7)	0.0174 (7)	0.0171 (7)	0.0021 (5)	0.0026 (5)	0.0028 (5)
C42	0.0226 (8)	0.0170 (7)	0.0216 (8)	0.0051 (6)	0.0077 (6)	0.0019 (6)
C43	0.0233 (8)	0.0140 (6)	0.0220 (8)	0.0041 (6)	0.0076 (6)	0.0027 (6)
C44	0.0236 (9)	0.0399 (11)	0.0258 (10)	0.0139 (8)	0.0018 (7)	0.0054 (8)
C45	0.0329 (11)	0.0289 (10)	0.0269 (10)	0.0046 (8)	0.0050 (8)	−0.0065 (8)
C46	0.0414 (13)	0.0302 (10)	0.0290 (10)	0.0113 (9)	0.0170 (9)	0.0019 (8)
C47	0.0185 (7)	0.0158 (6)	0.0161 (7)	0.0040 (5)	−0.0008 (6)	0.0024 (5)
C48	0.0180 (7)	0.0186 (7)	0.0185 (7)	0.0043 (6)	0.0004 (6)	0.0043 (6)
C49	0.0157 (7)	0.0194 (7)	0.0208 (8)	0.0044 (6)	0.0009 (6)	0.0065 (6)
C50	0.0153 (7)	0.0175 (7)	0.0160 (7)	0.0045 (5)	0.0017 (5)	0.0025 (5)
C51	0.0175 (7)	0.0188 (7)	0.0190 (7)	0.0066 (6)	0.0039 (6)	0.0036 (6)
C52	0.0171 (7)	0.0179 (7)	0.0179 (7)	0.0049 (6)	0.0022 (6)	0.0030 (6)
C53	0.0166 (7)	0.0194 (7)	0.0190 (7)	0.0020 (6)	0.0022 (6)	0.0027 (6)
C54	0.0194 (7)	0.0186 (7)	0.0170 (7)	0.0041 (6)	0.0032 (6)	0.0025 (6)
C55	0.0190 (7)	0.0147 (6)	0.0205 (8)	0.0040 (5)	0.0031 (6)	0.0035 (6)
Cl1	0.0647 (5)	0.0448 (4)	0.0421 (4)	0.0295 (3)	0.0040 (3)	−0.0036 (3)
C56	0.029 (2)	0.0228 (17)	0.035 (2)	0.0055 (15)	0.0140 (17)	0.0021 (15)

### Geometric parameters (Å, °)

**Table:**

Ru1—C48	1.8932 (18)	C15—C16	1.393 (3)
Ru1—C49	1.9365 (19)	C15—H15A	0.9300
Ru1—C47	1.9401 (19)	C16—C17	1.390 (3)
Ru1—P1	2.3280 (4)	C16—H16A	0.9300
Ru1—Ru2	2.84289 (18)	C17—C18	1.388 (3)
Ru1—Ru3	2.89756 (18)	C17—H17A	0.9300
Ru2—C51	1.9030 (18)	C18—C19	1.392 (3)
Ru2—C50	1.9313 (17)	C18—H18A	0.9300
Ru2—C52	1.9340 (18)	C19—H19A	0.9300
Ru2—P2	2.3212 (4)	C20—C21	1.391 (2)
Ru2—Ru3	2.81836 (19)	C20—C25	1.402 (2)
Ru3—C54	1.8770 (18)	C21—C22	1.398 (3)
Ru3—C53	1.9245 (19)	C21—H21A	0.9300
Ru3—C55	1.9445 (19)	C22—C23	1.382 (3)
Ru3—As1	2.4534 (2)	C22—H22A	0.9300
As1—C38	1.9413 (17)	C23—C24	1.388 (3)
As1—C26	1.9425 (17)	C23—H23A	0.9300
As1—C32	1.9459 (17)	C24—C25	1.385 (3)
P1—C7	1.8311 (17)	C24—H24A	0.9300
P1—C1	1.8381 (17)	C25—H25A	0.9300
P1—C13	1.8508 (17)	C26—C27	1.390 (2)
P2—C14	1.8202 (17)	C26—C31	1.396 (2)
P2—C20	1.8311 (17)	C27—C28	1.394 (3)
P2—C13	1.8391 (16)	C27—H27A	0.9300
S1—C29	1.7578 (18)	C28—C29	1.396 (3)
S1—C44	1.798 (2)	C28—H28A	0.9300
S2—C35	1.7571 (18)	C29—C30	1.398 (3)
S2—C45	1.789 (2)	C30—C31	1.389 (3)
S3—C41	1.7629 (18)	C30—H30A	0.9300
S3—C46	1.800 (2)	C31—H31A	0.9300
O1—C47	1.147 (2)	C32—C33	1.393 (3)
O2—C48	1.147 (2)	C32—C37	1.397 (2)
O3—C49	1.147 (2)	C33—C34	1.395 (3)
O4—C50	1.146 (2)	C33—H33A	0.9300
O5—C51	1.141 (2)	C34—C35	1.395 (3)
O6—C52	1.147 (2)	C34—H34A	0.9300
O7—C53	1.150 (2)	C35—C36	1.397 (3)
O8—C54	1.151 (2)	C36—C37	1.388 (3)
O9—C55	1.139 (2)	C36—H36A	0.9300
C1—C6	1.394 (3)	C37—H37A	0.9300
C1—C2	1.406 (3)	C38—C39	1.395 (2)
C2—C3	1.387 (3)	C38—C43	1.403 (2)
C2—H2A	0.9300	C39—C40	1.389 (2)
C3—C4	1.391 (4)	C39—H39A	0.9300
C3—H3A	0.9300	C40—C41	1.399 (3)
C4—C5	1.387 (3)	C40—H40A	0.9300
C4—H4A	0.9300	C41—C42	1.397 (2)
C5—C6	1.399 (3)	C42—C43	1.389 (3)
C5—H5A	0.9300	C42—H42A	0.9300
C6—H6A	0.9300	C43—H43A	0.9300
C7—C8	1.394 (2)	C44—H44A	0.9600
C7—C12	1.400 (2)	C44—H44B	0.9600
C8—C9	1.391 (3)	C44—H44C	0.9600
C8—H8A	0.9300	C45—H45A	0.9600
C9—C10	1.383 (3)	C45—H45B	0.9600
C9—H9A	0.9300	C45—H45C	0.9600
C10—C11	1.384 (3)	C46—H46A	0.9600
C10—H10A	0.9300	C46—H46B	0.9600
C11—C12	1.389 (3)	C46—H46C	0.9600
C11—H11A	0.9300	Cl1—C56	1.640 (5)
C12—H12A	0.9300	Cl1—C56^i^	1.763 (5)
C13—H13A	0.9700	C56—Cl1^i^	1.763 (5)
C13—H13B	0.9700	C56—H56A	0.9600
C14—C15	1.397 (2)	C56—H56B	0.9600
C14—C19	1.399 (2)		
			
C48—Ru1—C49	91.37 (8)	C18—C17—H17A	120.2
C48—Ru1—C47	91.58 (8)	C16—C17—H17A	120.2
C49—Ru1—C47	168.53 (7)	C17—C18—C19	120.21 (18)
C48—Ru1—P1	98.15 (6)	C17—C18—H18A	119.9
C49—Ru1—P1	94.01 (6)	C19—C18—H18A	119.9
C47—Ru1—P1	96.54 (5)	C18—C19—C14	120.53 (16)
C48—Ru1—Ru2	169.01 (6)	C18—C19—H19A	119.7
C49—Ru1—Ru2	93.12 (5)	C14—C19—H19A	119.7
C47—Ru1—Ru2	82.13 (5)	C21—C20—C25	118.92 (16)
P1—Ru1—Ru2	91.541 (11)	C21—C20—P2	123.14 (13)
C48—Ru1—Ru3	113.33 (5)	C25—C20—P2	117.93 (13)
C49—Ru1—Ru3	72.67 (5)	C20—C21—C22	120.29 (18)
C47—Ru1—Ru3	96.00 (5)	C20—C21—H21A	119.9
P1—Ru1—Ru3	145.643 (12)	C22—C21—H21A	119.9
Ru2—Ru1—Ru3	58.799 (4)	C23—C22—C21	120.34 (19)
C51—Ru2—C50	91.76 (7)	C23—C22—H22A	119.8
C51—Ru2—C52	90.74 (7)	C21—C22—H22A	119.8
C50—Ru2—C52	172.03 (7)	C22—C23—C24	119.62 (18)
C51—Ru2—P2	103.48 (6)	C22—C23—H23A	120.2
C50—Ru2—P2	90.49 (5)	C24—C23—H23A	120.2
C52—Ru2—P2	96.28 (5)	C25—C24—C23	120.51 (18)
C51—Ru2—Ru3	106.23 (6)	C25—C24—H24A	119.7
C50—Ru2—Ru3	92.76 (5)	C23—C24—H24A	119.7
C52—Ru2—Ru3	79.28 (5)	C24—C25—C20	120.31 (17)
P2—Ru2—Ru3	149.985 (12)	C24—C25—H25A	119.8
C51—Ru2—Ru1	164.61 (5)	C20—C25—H25A	119.8
C50—Ru2—Ru1	80.11 (5)	C27—C26—C31	119.21 (16)
C52—Ru2—Ru1	95.68 (5)	C27—C26—As1	123.23 (13)
P2—Ru2—Ru1	89.729 (11)	C31—C26—As1	117.13 (13)
Ru3—Ru2—Ru1	61.569 (5)	C26—C27—C28	120.64 (17)
C54—Ru3—C53	92.51 (8)	C26—C27—H27A	119.7
C54—Ru3—C55	92.73 (7)	C28—C27—H27A	119.7
C53—Ru3—C55	174.29 (7)	C27—C28—C29	120.00 (17)
C54—Ru3—As1	101.07 (6)	C27—C28—H28A	120.0
C53—Ru3—As1	90.73 (5)	C29—C28—H28A	120.0
C55—Ru3—As1	90.50 (5)	C28—C29—C30	119.40 (17)
C54—Ru3—Ru2	89.15 (6)	C28—C29—S1	124.59 (15)
C53—Ru3—Ru2	95.37 (5)	C30—C29—S1	116.01 (14)
C55—Ru3—Ru2	82.43 (5)	C31—C30—C29	120.22 (17)
As1—Ru3—Ru2	167.874 (7)	C31—C30—H30A	119.9
C54—Ru3—Ru1	144.62 (6)	C29—C30—H30A	119.9
C53—Ru3—Ru1	75.80 (5)	C30—C31—C26	120.48 (17)
C55—Ru3—Ru1	98.58 (5)	C30—C31—H31A	119.8
As1—Ru3—Ru1	112.165 (7)	C26—C31—H31A	119.8
Ru2—Ru3—Ru1	59.632 (5)	C33—C32—C37	118.60 (16)
C38—As1—C26	101.53 (7)	C33—C32—As1	121.12 (13)
C38—As1—C32	102.50 (7)	C37—C32—As1	120.26 (13)
C26—As1—C32	98.87 (7)	C32—C33—C34	120.79 (17)
C38—As1—Ru3	115.32 (5)	C32—C33—H33A	119.6
C26—As1—Ru3	122.36 (5)	C34—C33—H33A	119.6
C32—As1—Ru3	113.31 (5)	C33—C34—C35	120.21 (17)
C7—P1—C1	98.31 (8)	C33—C34—H34A	119.9
C7—P1—C13	102.25 (8)	C35—C34—H34A	119.9
C1—P1—C13	103.32 (8)	C34—C35—C36	119.18 (16)
C7—P1—Ru1	115.19 (6)	C34—C35—S2	124.52 (15)
C1—P1—Ru1	120.08 (6)	C36—C35—S2	116.29 (14)
C13—P1—Ru1	114.97 (5)	C37—C36—C35	120.20 (17)
C14—P2—C20	105.03 (8)	C37—C36—H36A	119.9
C14—P2—C13	105.42 (8)	C35—C36—H36A	119.9
C20—P2—C13	99.85 (8)	C36—C37—C32	121.00 (17)
C14—P2—Ru2	117.27 (6)	C36—C37—H37A	119.5
C20—P2—Ru2	117.45 (6)	C32—C37—H37A	119.5
C13—P2—Ru2	109.85 (6)	C39—C38—C43	118.98 (16)
C29—S1—C44	103.84 (10)	C39—C38—As1	120.45 (13)
C35—S2—C45	102.34 (10)	C43—C38—As1	120.47 (12)
C41—S3—C46	103.70 (10)	C40—C39—C38	120.25 (16)
C6—C1—C2	119.03 (16)	C40—C39—H39A	119.9
C6—C1—P1	121.32 (13)	C38—C39—H39A	119.9
C2—C1—P1	119.57 (14)	C39—C40—C41	120.81 (16)
C3—C2—C1	120.6 (2)	C39—C40—H40A	119.6
C3—C2—H2A	119.7	C41—C40—H40A	119.6
C1—C2—H2A	119.7	C42—C41—C40	118.98 (16)
C2—C3—C4	119.9 (2)	C42—C41—S3	124.37 (14)
C2—C3—H3A	120.0	C40—C41—S3	116.61 (13)
C4—C3—H3A	120.0	C43—C42—C41	120.27 (16)
C5—C4—C3	120.11 (19)	C43—C42—H42A	119.9
C5—C4—H4A	119.9	C41—C42—H42A	119.9
C3—C4—H4A	119.9	C42—C43—C38	120.66 (16)
C4—C5—C6	120.2 (2)	C42—C43—H43A	119.7
C4—C5—H5A	119.9	C38—C43—H43A	119.7
C6—C5—H5A	119.9	S1—C44—H44A	109.5
C1—C6—C5	120.17 (18)	S1—C44—H44B	109.5
C1—C6—H6A	119.9	H44A—C44—H44B	109.5
C5—C6—H6A	119.9	S1—C44—H44C	109.5
C8—C7—C12	118.82 (16)	H44A—C44—H44C	109.5
C8—C7—P1	124.27 (13)	H44B—C44—H44C	109.5
C12—C7—P1	116.80 (13)	S2—C45—H45A	109.5
C9—C8—C7	120.58 (17)	S2—C45—H45B	109.5
C9—C8—H8A	119.7	H45A—C45—H45B	109.5
C7—C8—H8A	119.7	S2—C45—H45C	109.5
C10—C9—C8	119.95 (18)	H45A—C45—H45C	109.5
C10—C9—H9A	120.0	H45B—C45—H45C	109.5
C8—C9—H9A	120.0	S3—C46—H46A	109.5
C9—C10—C11	120.15 (17)	S3—C46—H46B	109.5
C9—C10—H10A	119.9	H46A—C46—H46B	109.5
C11—C10—H10A	119.9	S3—C46—H46C	109.5
C10—C11—C12	120.19 (17)	H46A—C46—H46C	109.5
C10—C11—H11A	119.9	H46B—C46—H46C	109.5
C12—C11—H11A	119.9	O1—C47—Ru1	174.90 (15)
C11—C12—C7	120.31 (17)	O2—C48—Ru1	177.15 (17)
C11—C12—H12A	119.8	O3—C49—Ru1	171.53 (15)
C7—C12—H12A	119.8	O4—C50—Ru2	175.33 (16)
P2—C13—P1	114.22 (9)	O5—C51—Ru2	177.76 (17)
P2—C13—H13A	108.7	O6—C52—Ru2	174.50 (16)
P1—C13—H13A	108.7	O7—C53—Ru3	171.18 (16)
P2—C13—H13B	108.7	O8—C54—Ru3	174.27 (17)
P1—C13—H13B	108.7	O9—C55—Ru3	174.68 (16)
H13A—C13—H13B	107.6	C56—Cl1—C56^i^	60.6 (2)
C15—C14—C19	119.00 (16)	Cl1—C56—C56^i^	63.3 (3)
C15—C14—P2	122.11 (13)	Cl1—C56—Cl1^i^	119.4 (2)
C19—C14—P2	118.75 (13)	C56^i^—C56—Cl1^i^	56.1 (3)
C16—C15—C14	120.10 (17)	Cl1—C56—H56A	107.3
C16—C15—H15A	119.9	C56^i^—C56—H56A	126.2
C14—C15—H15A	119.9	Cl1^i^—C56—H56A	107.4
C17—C16—C15	120.56 (18)	Cl1—C56—H56B	107.5
C17—C16—H16A	119.7	C56^i^—C56—H56B	126.4
C15—C16—H16A	119.7	Cl1^i^—C56—H56B	107.4
C18—C17—C16	119.55 (17)	H56A—C56—H56B	107.2
			
C48—Ru1—Ru2—C51	−86.1 (4)	C7—P1—C1—C6	−128.05 (16)
C49—Ru1—Ru2—C51	27.9 (2)	C13—P1—C1—C6	127.18 (16)
C47—Ru1—Ru2—C51	−141.6 (2)	Ru1—P1—C1—C6	−2.49 (18)
P1—Ru1—Ru2—C51	122.0 (2)	C7—P1—C1—C2	48.68 (16)
Ru3—Ru1—Ru2—C51	−39.7 (2)	C13—P1—C1—C2	−56.08 (16)
C48—Ru1—Ru2—C50	−145.0 (3)	Ru1—P1—C1—C2	174.25 (13)
C49—Ru1—Ru2—C50	−31.07 (8)	C6—C1—C2—C3	−0.6 (3)
C47—Ru1—Ru2—C50	159.43 (7)	P1—C1—C2—C3	−177.41 (17)
P1—Ru1—Ru2—C50	63.04 (5)	C1—C2—C3—C4	0.5 (3)
Ru3—Ru1—Ru2—C50	−98.62 (5)	C2—C3—C4—C5	0.4 (4)
C48—Ru1—Ru2—C52	28.1 (3)	C3—C4—C5—C6	−1.1 (4)
C49—Ru1—Ru2—C52	142.10 (8)	C2—C1—C6—C5	−0.2 (3)
C47—Ru1—Ru2—C52	−27.41 (7)	P1—C1—C6—C5	176.61 (17)
P1—Ru1—Ru2—C52	−123.80 (5)	C4—C5—C6—C1	1.0 (3)
Ru3—Ru1—Ru2—C52	74.54 (5)	C1—P1—C7—C8	−97.59 (16)
C48—Ru1—Ru2—P2	124.4 (3)	C13—P1—C7—C8	8.06 (17)
C49—Ru1—Ru2—P2	−121.61 (6)	Ru1—P1—C7—C8	133.47 (14)
C47—Ru1—Ru2—P2	68.88 (5)	C1—P1—C7—C12	78.45 (15)
P1—Ru1—Ru2—P2	−27.510 (16)	C13—P1—C7—C12	−175.89 (13)
Ru3—Ru1—Ru2—P2	170.831 (12)	Ru1—P1—C7—C12	−50.48 (15)
C48—Ru1—Ru2—Ru3	−46.4 (3)	C12—C7—C8—C9	0.5 (3)
C49—Ru1—Ru2—Ru3	67.56 (5)	P1—C7—C8—C9	176.51 (14)
C47—Ru1—Ru2—Ru3	−101.95 (5)	C7—C8—C9—C10	−0.5 (3)
P1—Ru1—Ru2—Ru3	161.659 (12)	C8—C9—C10—C11	0.0 (3)
C51—Ru2—Ru3—C54	−27.64 (8)	C9—C10—C11—C12	0.6 (3)
C50—Ru2—Ru3—C54	−120.28 (7)	C10—C11—C12—C7	−0.5 (3)
C52—Ru2—Ru3—C54	59.99 (8)	C8—C7—C12—C11	0.0 (3)
P2—Ru2—Ru3—C54	143.95 (6)	P1—C7—C12—C11	−176.27 (15)
Ru1—Ru2—Ru3—C54	162.53 (6)	C14—P2—C13—P1	82.37 (10)
C51—Ru2—Ru3—C53	−120.08 (8)	C20—P2—C13—P1	−168.91 (9)
C50—Ru2—Ru3—C53	147.28 (7)	Ru2—P2—C13—P1	−44.85 (10)
C52—Ru2—Ru3—C53	−32.46 (8)	C7—P1—C13—P2	143.58 (9)
P2—Ru2—Ru3—C53	51.51 (6)	C1—P1—C13—P2	−114.69 (10)
Ru1—Ru2—Ru3—C53	70.08 (5)	Ru1—P1—C13—P2	18.03 (11)
C51—Ru2—Ru3—C55	65.23 (8)	C20—P2—C14—C15	−107.16 (15)
C50—Ru2—Ru3—C55	−27.42 (7)	C13—P2—C14—C15	−2.23 (17)
C52—Ru2—Ru3—C55	152.85 (7)	Ru2—P2—C14—C15	120.35 (14)
P2—Ru2—Ru3—C55	−123.19 (6)	C20—P2—C14—C19	77.37 (15)
Ru1—Ru2—Ru3—C55	−104.61 (5)	C13—P2—C14—C19	−177.69 (14)
C51—Ru2—Ru3—As1	120.05 (6)	Ru2—P2—C14—C19	−55.12 (15)
C50—Ru2—Ru3—As1	27.40 (6)	C19—C14—C15—C16	1.8 (3)
C52—Ru2—Ru3—As1	−152.33 (6)	P2—C14—C15—C16	−173.62 (15)
P2—Ru2—Ru3—As1	−68.37 (4)	C14—C15—C16—C17	0.1 (3)
Ru1—Ru2—Ru3—As1	−49.79 (3)	C15—C16—C17—C18	−1.7 (3)
C51—Ru2—Ru3—Ru1	169.84 (6)	C16—C17—C18—C19	1.3 (3)
C50—Ru2—Ru3—Ru1	77.19 (5)	C17—C18—C19—C14	0.7 (3)
C52—Ru2—Ru3—Ru1	−102.54 (5)	C15—C14—C19—C18	−2.3 (3)
P2—Ru2—Ru3—Ru1	−18.57 (2)	P2—C14—C19—C18	173.33 (14)
C48—Ru1—Ru3—C54	140.11 (11)	C14—P2—C20—C21	2.86 (17)
C49—Ru1—Ru3—C54	−136.05 (11)	C13—P2—C20—C21	−106.16 (16)
C47—Ru1—Ru3—C54	45.78 (11)	Ru2—P2—C20—C21	135.25 (14)
P1—Ru1—Ru3—C54	−65.11 (10)	C14—P2—C20—C25	−178.56 (13)
Ru2—Ru1—Ru3—C54	−31.24 (10)	C13—P2—C20—C25	72.41 (15)
C48—Ru1—Ru3—C53	66.27 (8)	Ru2—P2—C20—C25	−46.17 (15)
C49—Ru1—Ru3—C53	150.11 (8)	C25—C20—C21—C22	−0.1 (3)
C47—Ru1—Ru3—C53	−28.06 (8)	P2—C20—C21—C22	178.42 (15)
P1—Ru1—Ru3—C53	−138.95 (6)	C20—C21—C22—C23	−0.4 (3)
Ru2—Ru1—Ru3—C53	−105.08 (6)	C21—C22—C23—C24	0.2 (3)
C48—Ru1—Ru3—C55	−112.70 (8)	C22—C23—C24—C25	0.5 (3)
C49—Ru1—Ru3—C55	−28.86 (8)	C23—C24—C25—C20	−1.1 (3)
C47—Ru1—Ru3—C55	152.97 (7)	C21—C20—C25—C24	0.9 (3)
P1—Ru1—Ru3—C55	42.07 (6)	P2—C20—C25—C24	−177.73 (14)
Ru2—Ru1—Ru3—C55	75.95 (5)	C38—As1—C26—C27	−12.47 (16)
C48—Ru1—Ru3—As1	−18.62 (6)	C32—As1—C26—C27	92.33 (16)
C49—Ru1—Ru3—As1	65.21 (6)	Ru3—As1—C26—C27	−142.70 (13)
C47—Ru1—Ru3—As1	−112.95 (5)	C38—As1—C26—C31	175.20 (13)
P1—Ru1—Ru3—As1	136.15 (2)	C32—As1—C26—C31	−80.00 (14)
Ru2—Ru1—Ru3—As1	170.025 (8)	Ru3—As1—C26—C31	44.97 (15)
C48—Ru1—Ru3—Ru2	171.35 (6)	C31—C26—C27—C28	1.5 (3)
C49—Ru1—Ru3—Ru2	−104.81 (6)	As1—C26—C27—C28	−170.72 (14)
C47—Ru1—Ru3—Ru2	77.02 (5)	C26—C27—C28—C29	0.9 (3)
P1—Ru1—Ru3—Ru2	−33.88 (2)	C27—C28—C29—C30	−2.4 (3)
C54—Ru3—As1—C38	68.63 (8)	C27—C28—C29—S1	178.11 (15)
C53—Ru3—As1—C38	161.33 (8)	C44—S1—C29—C28	−8.66 (19)
C55—Ru3—As1—C38	−24.24 (7)	C44—S1—C29—C30	171.81 (15)
Ru2—Ru3—As1—C38	−78.36 (6)	C28—C29—C30—C31	1.5 (3)
Ru1—Ru3—As1—C38	−123.72 (5)	S1—C29—C30—C31	−178.91 (15)
C54—Ru3—As1—C26	−167.21 (8)	C29—C30—C31—C26	0.8 (3)
C53—Ru3—As1—C26	−74.51 (8)	C27—C26—C31—C30	−2.3 (3)
C55—Ru3—As1—C26	99.91 (8)	As1—C26—C31—C30	170.35 (14)
Ru2—Ru3—As1—C26	45.79 (7)	C38—As1—C32—C33	82.51 (16)
Ru1—Ru3—As1—C26	0.44 (6)	C26—As1—C32—C33	−21.48 (16)
C54—Ru3—As1—C32	−49.05 (8)	Ru3—As1—C32—C33	−152.57 (13)
C53—Ru3—As1—C32	43.65 (8)	C38—As1—C32—C37	−99.25 (15)
C55—Ru3—As1—C32	−141.93 (7)	C26—As1—C32—C37	156.76 (15)
Ru2—Ru3—As1—C32	163.95 (6)	Ru3—As1—C32—C37	25.67 (16)
Ru1—Ru3—As1—C32	118.59 (5)	C37—C32—C33—C34	−0.1 (3)
C48—Ru1—P1—C7	77.35 (8)	As1—C32—C33—C34	178.20 (14)
C49—Ru1—P1—C7	−14.62 (8)	C32—C33—C34—C35	0.9 (3)
C47—Ru1—P1—C7	169.89 (8)	C33—C34—C35—C36	−1.2 (3)
Ru2—Ru1—P1—C7	−107.85 (6)	C33—C34—C35—S2	178.57 (15)
Ru3—Ru1—P1—C7	−79.37 (7)	C45—S2—C35—C34	−8.56 (19)
C48—Ru1—P1—C1	−39.84 (9)	C45—S2—C35—C36	171.24 (16)
C49—Ru1—P1—C1	−131.80 (8)	C34—C35—C36—C37	0.8 (3)
C47—Ru1—P1—C1	52.71 (8)	S2—C35—C36—C37	−179.06 (15)
Ru2—Ru1—P1—C1	134.96 (7)	C35—C36—C37—C32	0.1 (3)
Ru3—Ru1—P1—C1	163.45 (6)	C33—C32—C37—C36	−0.4 (3)
C48—Ru1—P1—C13	−164.13 (8)	As1—C32—C37—C36	−178.69 (15)
C49—Ru1—P1—C13	103.91 (8)	C26—As1—C38—C39	120.99 (14)
C47—Ru1—P1—C13	−71.58 (8)	C32—As1—C38—C39	19.08 (16)
Ru2—Ru1—P1—C13	10.67 (6)	Ru3—As1—C38—C39	−104.52 (14)
Ru3—Ru1—P1—C13	39.16 (7)	C26—As1—C38—C43	−62.55 (16)
C51—Ru2—P2—C14	110.73 (8)	C32—As1—C38—C43	−164.46 (15)
C50—Ru2—P2—C14	−157.34 (8)	Ru3—As1—C38—C43	71.94 (15)
C52—Ru2—P2—C14	18.45 (8)	C43—C38—C39—C40	−1.3 (3)
Ru3—Ru2—P2—C14	−60.97 (7)	As1—C38—C39—C40	175.19 (14)
Ru1—Ru2—P2—C14	−77.23 (6)	C38—C39—C40—C41	2.0 (3)
C51—Ru2—P2—C20	−15.90 (8)	C39—C40—C41—C42	−0.8 (3)
C50—Ru2—P2—C20	76.03 (8)	C39—C40—C41—S3	−178.72 (14)
C52—Ru2—P2—C20	−108.18 (8)	C46—S3—C41—C42	14.55 (19)
Ru3—Ru2—P2—C20	172.40 (6)	C46—S3—C41—C40	−167.69 (15)
Ru1—Ru2—P2—C20	156.14 (6)	C40—C41—C42—C43	−1.1 (3)
C51—Ru2—P2—C13	−129.00 (8)	S3—C41—C42—C43	176.65 (15)
C50—Ru2—P2—C13	−37.07 (8)	C41—C42—C43—C38	1.8 (3)
C52—Ru2—P2—C13	138.72 (8)	C39—C38—C43—C42	−0.6 (3)
Ru3—Ru2—P2—C13	59.30 (7)	As1—C38—C43—C42	−177.08 (15)
Ru1—Ru2—P2—C13	43.03 (6)		

Symmetry codes: (i) −*x*+1, −*y*, −*z*+1.

### Hydrogen-bond geometry (Å, °)

**Table:**

Cg1, Cg2, Cg3 and Cg4 are the centroids of the C14–C19, C26–C31, C1–C6 and C32–C37 benzene rings, respectively.

**Table:**

*D*—H···*A*	*D*—H	H···*A*	*D*···*A*	*D*—H···*A*
C46—H46C···O4^ii^	0.96	2.59	3.401 (3)	142
C11—H11A···Cg1^iii^	0.93	2.91	3.640 (2)	137
C18—H18A···Cg2^iv^	0.93	2.96	3.652 (2)	133
C36—H36A···Cg3^v^	0.93	2.81	3.687 (2)	157
C45—H45B···Cg4^vi^	0.96	2.90	3.760 (3)	150

Symmetry codes: (ii) −*x*+1, −*y*+2, −*z*+1; (iii) *x*−1, *y*, *z*; (iv) *x*+1, *y*, *z*; (v) *x*, *y*−1, *z*; (vi) −*x*+1, −*y*+1, −*z*.
